# Social camouflaging predicts eating disorder symptomatology among female patients with Borderline Personality Disorder

**DOI:** 10.3389/fpsyt.2025.1646586

**Published:** 2025-10-10

**Authors:** Barbara Carpita, Benedetta Nardi, Federico Giovannoni, Francesca Parri, Cristiana Pronestì, Chiara Bonelli, Gabriele Massimetti, Stefano Pini, Antonio Bruno, Maria Rosaria Anna Muscatello, Bianca Della Rocca, Andrea Fiorillo, Liliana Dell’Osso

**Affiliations:** ^1^ Department of Clinical and Experimental Medicine, Section of Psychiatry, University of Pisa, Pisa, Italy; ^2^ Department of Biomedical and Dental Sciences and Morphofunctional Imaging, University of Messina, Messina, Italy; ^3^ Department of Psychiatry, University of Campania “Luigi Vanvitelli”, Naples, Italy

**Keywords:** camouflaging, eating disorders, Borderline Personality Disorder, autistic traits, disordered eating

## Abstract

**Background:**

Borderline Personality Disorder (BPD) frequently co-occurs with both eating disorders (EDs) and elevated autistic traits, particularly among women. Social camouflaging—a set of strategies used to mask neurodivergent behaviors—has been extensively studied in autism spectrum conditions but remains underexplored in BPD populations. Given emerging evidence linking camouflaging behaviors to disordered eating, this study aimed to investigate the relationship between social camouflaging and eating disorder symptomatology in women with BPD.

**Methods:**

A total of 110 female participants (64 with BPD, 46 healthy controls) were assessed using the Camouflaging Autistic Traits Questionnaire (CAT-Q), the Eating Disorder Inventory-2 (EDI-2), and the Adult Autism Subthreshold Spectrum (AdAS Spectrum).

**Results:**

Women with BPD scored significantly higher on all domains of the CAT-Q, AdAS Spectrum, and EDI-2. Significant correlations emerged between CAT-Q and EDI-2 scores. CAT-Q total score and CAT-Q compensation domain significantly predicted EDI-2 total score.

**Conclusion:**

Social camouflaging is significantly elevated in women with BPD and is closely associated with disordered eating symptoms. Findings highlight camouflaging as a potential transdiagnostic mechanism linking autistic traits and ED symptomatology in BPD populations, with implications for diagnostic clarity and personalized treatment strategies.

## 1 Introduction

Borderline Personality Disorder (BPD) is a complex and multifaceted mental health condition characterized by pervasive instability in mood, self-image, interpersonal relationships, and impulse control (1). Individuals with BPD frequently struggle with emotional dysregulation, impulsivity and self-harming behaviors, identity disturbances, and profound fear of abandonment (1). BPD is estimated to affect between 0.7% and 2.7% of the general population, with markedly higher rates observed in clinical settings (up to 22% among psychiatric inpatients) (2). The disorder is often associated with profound functional impairments and elevated rates of comorbid psychiatric conditions (3). Although BPD is diagnosed more frequently in women, with a clinical gender ratio of roughly 3:1, this discrepancy may be influenced by gendered diagnostic practices and sociocultural biases in both symptom expression and referral patterns (1, 3). Recent evidences are suggesting how the core symptoms of BPD are often rooted in a history of trauma. Experiences such as abandonment, neglect, and various forms of abuse—particularly physical and sexual—are common among those affected and may contribute significantly to the disorder’s development and clinical course (4–6). Moreover, BPD often coexists with other psychiatric disorders, among which eating disorders are especially prevalent. Conditions such as anorexia nervosa (AN), bulimia nervosa (BN), and binge-eating disorder (BED) are not only commonly comorbid with BPD but also disproportionately affect women, underscoring the complex interplay of gender, trauma, and psychopathology in this population (7, 8).

Recent research has increasingly highlighted the significant overlap, especially in female populations, between BPD and features typically associated with Autism Spectrum Disorder (ASD), a neurodevelopmental disorder characterized by deficits in social communication and the presence of restricted interests and repetitive behaviors (1, 9–12). This overlap includes traits such as social communication difficulties, heightened emotional sensitivity, and rigid thinking patterns (9, 10). Indeed, individuals with either condition may exhibit intense yet unstable interpersonal relationships, difficulties in both verbal and non-verbal communication, a tendency to act impulsively rather than verbalize emotions, impairments in Theory of Mind, emotional dysregulation, and engagement in self-harming behaviors (13–17). However, despite these similarities, important distinctions remain between the two.

One phenomenon that may bridge these conditions is social camouflaging—a set of compensatory strategies employed by individuals, often women, to mask neurodivergent traits and conform to neurotypical social expectations. Indeed, autistic individuals who do not present with intellectual impairments often demonstrate a greater awareness of their social communication challenges (18). In particular, this feature seems to be particularly present among female patients: some authors even hypothesized that the higher tendency to adopt social camouflaging strategies may contribute to an underdiagnosis of ASD among females (8, 19–22). This heightened self-awareness may drive them to consciously modify their behavior in order to align with the social norms and expectations of neurotypical individuals (14, 16). Within this context, the concept of social camouflaging has emerged as a significant area of interest in both clinical practice and research (19, 20, 23–25). Social camouflaging refers to a set of adaptive strategies aimed at concealing social difficulties and presenting oneself in a socially acceptable manner by subjects in the autism spectrum. These strategies can involve suppressing behaviors perceived as atypical or deliberately performing actions that are considered more socially normative in an effort to appear socially competent (19). Such behaviors are often acquired through observation of peers or intentional learning from various media sources. Examples include maintaining eye contact, mimicking facial expressions and body language, or using rehearsed phrases and scripted dialogue during interactions (19, 20). Notably, camouflaging can become increasingly sophisticated over time, especially when initiated in early childhood. While some aspects of these strategies may develop unconsciously, prolonged and consistent use may eventually contribute to the creation of a socially constructed persona, often referred to as a “mask,” which the individual adopts in public or interpersonal contexts (24). However, while social camouflaging has been well-documented in ASD, studies in different samples, such as university students, seem to point out a continuous distribution of camouflaging from the general to the clinical population, following the continuous distribution of autistic traits (25). However, its presence and clinical significance in BPD remain underexplored, despite the presence of high autistic features in BPD subjects according to the scientific literature (13).

In addition, as reported above, BPD patients frequently exhibit comorbid symptoms of eating disorders, further complicating diagnostic clarity and treatment planning. In fact, a growing body of research has shown that individuals with BPD frequently engage in disordered eating behaviors, even in the absence of a formal eating disorder diagnosis (26). These behaviors commonly include binge eating, restrictive dieting, meal skipping, and self-induced vomiting. Vice versa, a recent systematic review identified BPD as the most prevalent comorbid personality disorder among individuals exhibiting pathological eating behaviors (27). On the other hand, numerous investigations have documented a significant overlap between autism spectrum and eating disorders, with elevated levels of autistic traits consistently observed in individuals with AN and BN. While it could be hypothesized that autistic traits may at least in part constitute a common ground for different psychopathological manifestations such as BPD and eating disorders, recent studies also highlighted a possible association between camouflaging behaviors and eating disorder symptoms in ASD subjects (28–34). Some scholars have also proposed that specific presentations of BPD and eating disorders—particularly among females—may represent an alternative manifestation of the autism spectrum, underscoring the importance of considering neurodevelopmental dimensions in these clinical profiles (8).

The present study aims to fill a critical gap in the literature by examining the presence of social camouflaging among women diagnosed with BPD and its psychopathological correlates, specifically focusing on eating disorder symptomatology.

## 2 Materials and methods

### 2.1 Study sample and procedures

The study included a sample of 64 in and out patients diagnosed with BPD, followed at the Psychiatric clinic of the Azienda Ospedaliero-Universitaria of Pisa, as well as 46 healthy controls (HCs) recruited from medical and paramedical staff on a voluntary basis. Diagnosis were made through clinical assessment by trained psychiatrists. Patients recruited had not received a diagnosis of autism in childhood. Participants were excluded if they had intellectual disabilities, poor cooperation skills, were under 18 years of age, had language or intellectual impairments that could hinder the completion of assessments, or presented with ongoing psychotic symptoms. The study adhered to the principles outlined in the Declaration of Helsinki, and all recruitment and evaluation procedures were approved by the Ethics Committee of the Azienda Ospedaliero-Universitaria of Pisa (protocol code: 23910_Dell’osso; date of approval: 20th April 2023). Informed consent was obtained from all participants after a detailed explanation of the study, with an opportunity to ask questions. No financial compensation was provided for participation.

All participants underwent evaluation using the Adult Autism Subthreshold Spectrum (AdAS Spectrum), the Eating Disorder Inventory—2 (EDI-2) and the Camouflaging Autistic Traits Questionnaire (CAT-Q).

All questionnaires were administered in a quiet outpatient clinical setting at the Department of Psychiatry, University of Pisa. Participants completed the instruments in paper-and-pencil format in a dedicated room free from external distractions. The assessments were carried out during a single session, lasting approximately 60–90 minutes. A trained clinical psychiatry resident was present to provide standardized instructions, ensure comprehension of the items, and supervise the completion process. Participants were encouraged to ask clarification questions if needed, but no guidance on item content or interpretation was provided. Breaks were allowed to prevent fatigue, particularly for BPD patients who might experience distress when completing long questionnaires.

### 2.2 Measures

#### 2.2.1 The CAT-Q

The CAT-Q is a self-report instrument designed to evaluate camouflaging behaviors related to the autism spectrum in both clinical and non-clinical populations. It consists of 25 items, categorized into three domains: Assimilation, Masking, and Compensation, each assessed on a seven-point Likert scale. The Italian version of the questionnaire demonstrated excellent internal consistency, with a Cronbach’s alpha of 0.904. Moreover, it exhibited strong test-retest reliability and convergent validity when compared to other established autism assessment tools (35, 36).

#### 2.2.2 The EDI-2

The EDI-2 is a self-report tool designed to assess eating disorder symptoms and associated psychological traits. It comprises 91 items across 11 domains: Drive for Thinness, Bulimia, Body Dissatisfaction, Ineffectiveness, Perfectionism, Interpersonal Distrust, Interoceptive Awareness, Maturity Fears, Asceticism, and Impulsivity. Responses are given on a 6-point Likert scale. This broad range of sub-scales allows for a comprehensive evaluation of both behavioral and psychological aspects of eating disorders (37).

#### 2.2.3 The AdAS spectrum

The AdAS Spectrum is a 160-item self-report questionnaire designed to assess a wide range of autism-related symptoms in individuals without cognitive or language impairments. It covers seven domains: Childhood and adolescence, Verbal and Non-verbal communication, Empathy, a Rigidity and adherence to routine, Restricted interests and rumination, and Hyper- or Hyporeactivity to sensory stimuli. The validation study demonstrated strong internal consistency, excellent test-retest reliability, and convergent validity with other dimensional autism spectrum measures (38).

### 2.3 Statistical analysis

Cronbach’s Alpha were calculated for all psychometric questionnaires. Student t-test was used for comparing mean age. Mann-Whitney tests were used for comparing CAT-Q domains and total scores, as well as AdAS Spectrum and EDI-2 total scores among the two diagnostic groups.

Spearman’s correlation coefficients were then used to examine the relationships between CAT-Q and EDI-2 scores.

Afterwards, a linear regression analysis was performed using EDI-2 total score as dependent variable and CAT-Q total score and diagnostic group (BPD *vs* HC) as independent variables in order to investigate the eventual role of social camouflaging as a statistical predictor of greater symptoms of eating disorders. A second linear regression analysis was then performed with the same dependent variable and CAT-Q domain scores and diagnostic group as independent variables.

All statistical analyses were performed with SPSS version 26.0.

## 3 Results

The total sample was made of 110 female subjects of which 64 with a clinical diagnosis of BPD (mean age: 30.8 ± 12.17 years) and 46 healthy controls (mean age: 33.72 ± 10.09). The groups did not significantly differ for age. All questionnaires reported excellent Cronbach’s alpha values in the sample (Cronbach’s alpha for AdAS Spectrum:.978; for CAT-Q:.963; for EDI-2:.957) (see **Table 1**).

**Table 1:** Age comparison between diagnostic groups.

	HCs *mean ± SD*	BPD *mean ± SD*	t	p
Age	33.72 ± 10.09	30.80 ± 12.17	1.331	.186

The results of the Mann-Whitney tests revealed that individuals with BPD scored significantly higher across all CAT-Q domains, as well as in the total CAT-Q score. Additionally, they reported significantly higher scores on the AdAS Spectrum and EDI-2 total scores (see **Table 2**).

**Table 2:** Comparison of AdAS Spectrum, CAT-Q, EDI-2 and TALS scores among HCs and BPD.

	HCs *mean ± SD, mean rank*	BPD *mean ± SD, mean rank*	U	p
AdAS Spectrum				
AdAS Spectrum total score	21.93 ± 18.01, 26.10	76.47 ± 24.87, 76.60	2822.50	<.001*
CAT-Q				
Compensation	13.76 ± 7.40, 29.96	29.67 ± 11.28, 73.86	2647.00	<.001*
Masking	15.65 ± 9.02, 28.13	34.75 ± 8.12, 74.62	2685.00	<.001*
Assimilation	12.74 ± 7.24, 26.93	32.43 ± 9.89, 74.95	2694.00	<.001*
CAT-Q total score	42.15 ± 22.03, 26.87	96.81 ± 24.39, 75.00	2697.00	<.001*
EDI-2				
EDI-2 total score	33.52 ± 20.66, 32.62	79.61 ± 39.58, 71.95	26524.50	<.001*

*Significant values for p<.008(.05/6) if considering Bonferroni correction

The results of the correlation analysis showed a pattern of medium to strong correlations between EDI-2 and CAT-Q scores. Specifically, while CAT-Q and EDI-2 total scores were significantly and positively correlated, significant positive correlations were also found between almost all CAT-Q and EDI-2 domains, with the exception of *EDI-2 Interpersonal distrust* and *Social insecurity* domains, which were not significantly correlated with CAT-Q scores (see **Table 3**).

**Table 3:** Spearman’s correlations coefficients among AdAS Spectrum domains and total score and CAT-Q domains and total score in the total sample.

	Compensation	Masking	Assimilation	CAT-Q total score
Drive for Thinness	.438**	.439**	.406**	.446**
Bulimia	.441**	.434**	.337**	.411**
Body Dissatisfaction	.431**	.483**	.403**	.467**
Ineffectiveness	.495**	.564**	.510**	.572**
Perfectionism	.518**	.542**	.533**	.589**
Interpersonal Distrust	.165	.211	.226	.241
Interoceptive Awareness	.494**	.487**	.511**	.534**
Maturity Fears	.471**	.418**	.533**	.518**
Asceticism	.648**	.670**	.644**	.697**
Impulsivity	.658**	.646**	.649**	.704**
Social Insecurity	.075	.149	.222	.193
EDI-2 total score	. 652**	.695**	.665**	.721**

**Significant for p<.001 (.05/48) considering Bonferroni correction

Results from the linear regression analysis performed using EDI-2 total score as dependent variable and CAT-Q total score and diagnostic group as independent variables showed that the CAT-Q total score, but not diagnostic group, was significant positive predictors of greater eating disorder symptoms (see **Table 4**).

**Table 4:** Linear regression analysis with EDI-2 total score as dependent variable and CAT-Q total score and diagnostic group (BPD ***vs*** HC) as independent variables, carried in the whole sample.

	B(S.E.)	BETA	t	p	VIF
*constant*	2.054 (6.468)		.318	.751	
CAT-Q total score	.747 (.117)	.667	6.364	<.001	2.357
BPD diagnosis (absence/presence)	5.001 (8.449)	.062	.592	.555	2.357

R^2^, .715; Adjusted R^2^, .511; *Significant for p<.05

Results from a further linear regression analysis performed using EDI-2 total score as dependent variable and CAT-Q domains and diagnostic group as independent variables showed that CAT-Q *Compensation* domain was a significant positive predictor of greater eating disorder symptoms (see **Table 5**).

**Table 5:** Linear regression analysis with EDI-2 total score as dependent variable and CAT-Q domain scores and diagnostic group (BPD ***vs*** HC) as independent variables, carried in the whole sample.

	B(S.E.)	BETA	t	p	VIF
*constant*	2.867 (6.733)		.426	.671	
CAT-Q Compensation	.915 (.387)	.288	2.366	.020	3.126
CAT-Q Masking	.548 (.446)	.173	1.228	.222	4.175
CAT-Q Assimilation	.745 (.434)	.245	1.714	.089	4.309
BPD diagnosis (absence/presence)	6.082 (8.819)	.075	.690	.492	2.526

## 4 Discussion

The main objective of our study was to investigate the presence and correlates of camouflaging behaviors in a sample of patients with BPD, specifically focusing on eating disorder symptoms. As expected, BPD subjects scored significantly higher in the AdAS Spectrum, EDI-2 and CAT-Q questionnaires. The strong link between BPD and eating disorders is well-recognized (39). Numerous studies have shown that individuals with BPD often engage in disordered eating behaviors, such as binge eating, dieting, meal skipping, and vomiting, regardless of whether they have a formal eating disorder diagnosis (26). Additionally, some researchers have highlighted a direct relationship between these two conditions, suggesting that shared transdiagnostic factors, such as socio-cultural influences, common underlying causes, and overlapping symptoms —like emotional regulation difficulties, interpersonal struggles, self-concept issues, and impulsivity— play a role in both disorders (40). Also, the evidence of higher autistic traits in BPD patients is consistent with the current literature highlighting not only a greater presence of autistic symptomatology in patients with BPD, but also a partial clinical overlap between these two conditions (9–12, 41). Indeed, recent data have reported how BPD patients have an increased odds ratio of having an ASD diagnosis and vice versa a significant prevalence of BPD – up to 15% – in autistic subjects, supporting the hypothesis that disorders such as BPD can mask the presence of ASD, especially in women, leading to a greater rate of misdiagnosis (8, 9, 41–43). In this regard, the concept of a “female autistic phenotype” has long been acknowledged, referring to a distinct manifestation of autism in women with respect to the traditional, male-centered models of ASD (44–46).

A key feature of this phenotype is the ability to “camouflage” social difficulties across different social situations (47). Studies consistently show that women with full-blown ASD or elevated autistic traits often exhibit higher social motivation and a greater ability to engage in typical social connections (47, 48), allowing them to develop masking strategies to conceal their neuroatypical traits and better integrate with neurotypical individuals. This documented ability to camouflage in women with high autistic traits supports our finding of a higher score BPD group in both AdAS Spectrum and CAT-Q questionnaires, aimed at investigating autistic-like camouflaging behaviors.

Moreover, EDI-2 total score was significantly correlated with all CAT-Q domains and its total score, with CAT-Q total score also being a positive predictor of higher symptoms of eating disorders, without a predictive role of the diagnostic group. These results align with previous studies indicating a link between eating disorders and camouflaging behaviors (34, 49). Specifically, research by Bradley et al. highlighted that social camouflaging, as assessed by the CAT-Q, can predict symptoms of eating disorders in population with autism spectrum (34). In particular, the study suggests that camouflaging may contribute to the development of eating disorder symptoms, serving as a coping mechanism for social pressures. An intriguing aspect of this is the pivotal role that identity plays in the onset and persistence of these disorders (1, 50). Moreover, a more recent research how in a sample of university students camouflaging behaviors seem to mediate the relationship between autistic traits and orthorexic symptoms (1). Some have argued that eating disorders may stem from identity struggles, which are also one of the core issues in BPD. In this context, an eating disorder may provide a sense of self or a community to belong to (50). In addition, it is also possible that female subjects in the autism spectrum may try to focus on socially accepted interests among girls, such as eating styles and body shape, as an attempt to mask their autism; however, they may pursue the interest with the inflexible and pervasive fashion typical of autism spectrum, enhancing the risk to develop an eating disorder (1). Our results are in line with these data, suggesting an association between camouflaging and eating disorders in different populations. These findings add to the broader body of research showing that camouflaging behaviors are strongly associated with poor mental health outcomes, including symptoms of eating disorders (20, 34, 51).

Interestingly, the EDI-2 domains of *Ineffectiveness, Perfectionism, Interoceptive Awareness*, *Impulsivity* and *Ascetism* were those for which the strongest correlations were reported with CAT-Q scores. Although to this date there is paucity of literature on the topic, it is plausible that *Ineffectiveness*, conceptualized as feelings of low self-esteem, heightened sensitivity to criticism, and a sense of inadequacy, may be linked to the use of camouflaging behaviors in order to conceal the perceived social difficulties. Moreover, using such behaviors could eventually make the subject feel as though they are living a “double” or “false” life, which may lead to a sense of inadequacy and mental or physical exhaustion. This is partly because they often view their social acceptance as dependent on their ability to mask their true selves, rather than being accepted for who they are (20, 51–54). As for ascetism, camouflaging can contribute to the development of ascetic traits when an individual feels compelled to suppress or sacrifice parts of themselves in order to conform to social norms or expectations. Since camouflaging involves hiding or downplaying one’s behaviors and emotions to fit in or avoid social judgment, constant engagement in this process may lead a person to view their emotional, social, or physical needs as something to be repressed or ignored. This can foster a form of asceticism, where the individual attempts to “purify” themselves by voluntarily sacrificing their well-being or needs to meet an external standard of perfection or social acceptability. Over time, the suppression of emotions deemed “inappropriate” may cause them to perceive their needs or desires as unnecessary or even negative. Consequently, they may begin to reject and suppress these needs, viewing self-sacrifice as a virtue that helps them gain greater control or better adapt socially (22, 24, 55).

Concerning perfectionism, it could be hypothesized that these subjects, due to their heightened sensitivity to criticism and judgment, impose unrealistic standards on themselves. As a result, they often adopt communication strategies to compensate for their perceived social shortcomings and present an image of themselves that they believe is better and more socially acceptable. However, the idealized and perfect image they strive to project is often unattainable and unsustainable, which can lead to physical and mental exhaustion over time (19, 21, 54, 55). Altered interoceptive awareness has been widely documented in BPD populations, where it has frequently been associated with alexithymia and trauma-related dissociative symptoms (56–60). This difficulty in understanding and processing bodily sensations may lead to the adoption camouflaging behaviors in order to fit in with others, without basing on self-perceived stimuli (60–63). This hypothesis is further supported by a recent study that highlighted how interoceptive deficits correlated with measures of camouflaging (63). Lastly, the link between impulsivity and camouflaging likely stems from the emotional toll and build up stress and frustration of continually using strategies to conceal social difficulties. Individuals who engage in camouflaging often become deeply focused on hiding their social struggles to fit in or meet societal expectations. This ongoing effort can lead to significant emotional strain over time (21, 22, 64). While the fear of emotional dysregulation may enhance the drive for adopting compensation strategy in order to regulate emotion and properly behave in social situations, when emotional regulation falters, this accumulated pressure may trigger intense emotional reactions or impulsive behaviors. This also aligns with previous research indicating that camouflaging can predict emotional dysregulation in autistic individuals, with emotional regulation difficulties potentially mediating the link between social camouflaging and symptoms of depression or anxiety (65, 66).

Significant correlations were also found between CAT-Q and EDI-2 *Drive for thinness*, *Body dissatisfaction, Bulimia* and *Maturity Fears* domains. Although there is no systematic data on this, it is conceivable that individuals who have a strong drive for thinness may use masking to hide their emotional distress, such as feelings of shame, anxiety, or insecurity about their bodies. In this vein, masking may also involve hiding real emotions related to food, body image, or self-esteem. Similarly, when individuals experience dissatisfaction with their bodies, they may become hyper-aware of how others perceive them, and the fear of being judged or criticized based on their appearance may trigger masking behaviors, in which they try to hide their real emotions or discomfort (67, 68). On the other hand, bulimia is in fact often associated with intense feelings of shame, guilt and embarrassment for eating behaviors, such as binge eating and purging behaviors (through vomiting, excessive exercise or use of laxatives), which are considered socially inappropriate and unacceptable (69). It is therefore possible that to avoid this potential judgment, individuals with bulimia adopt camouflaging behaviors, which could include lying about their eating habits. On the other hand, it is possible that the habit of camouflaging may also enhance in these patients the risk to develop bulimia symptoms by try to maladaptively employ compensative behaviors also in other contexts, such as after binge eating, thus masking and compensating for the episode of loss of control as they try to do with autism symptoms.

Intriguingly, our results did not shown a correlation between EDI-2 interpersonal distrust and social insecurity domains and the CAT-Q. It is possible that the use of camouflaging strategies, although requiring a great mental effort and identity struggles, would allow subjects to feel relatively more confident in social situations, thus reducing insecurity feelings with respect to those with similar psychopathological traits who do not adopt camouflaging strategies (22, 24, 55, 70).

Our findings also bear potential clinical implications. Although preliminary, they suggest that the assessment of social camouflaging in women with BPD may enrich clinical evaluation and treatment planning. In particular, the combined use of the CAT-Q, together with dimensional measures such as the AdAS Spectrum and the EDI-2, could represent a useful battery for identifying patients in whom camouflaging strategies contribute to diagnostic complexity and symptom severity. From a therapeutic perspective, greater clinical awareness of camouflaging may help clinicians to explore masking behaviors that exacerbate emotional dysregulation and maladaptive eating patterns. Psychoeducation about camouflaging, the integration of emotion regulation skills training (as in dialectical behavior therapy), and interventions aimed at enhancing interoceptive awareness may represent promising strategies to address these processes. Furthermore, recognizing camouflaging may reduce the risk of misdiagnosing autistic traits in BPD patients and favor more personalized treatment approaches. Nevertheless, these considerations remain exploratory, and further longitudinal and interventional studies are warranted to provide evidence-based clinical guidelines.

These findings should be considered in the context of several limitations. Firstly, the study’s cross-sectional design limits our ability to draw conclusions about cause-and-effect relationships or the temporal progression of the variables being examined. Additionally, the reliance on self-report psychometric questionnaires to assess participants may lead to either over- or under-estimation of symptoms, depending on individuals’ subjective perceptions. Moreover, the relatively small size of the sample and the choice of an all-female sample limits the generalizability of the findings, making it challenging to apply these findings to larger or more diverse populations. The relatively small sample size also prevented us from performing more in-depth within-group analyses and stratifying patients for autistic traits severity.

## Data Availability

The original contributions presented in the study are included in the article/supplementary material. Further inquiries can be directed to the corresponding author.
